# Determining factors for the prevalence of depressive symptoms among postpartum mothers in lowland region in southern Nepal

**DOI:** 10.1371/journal.pone.0245199

**Published:** 2021-01-22

**Authors:** Devendra Raj Singh, Dev Ram Sunuwar, Shraddha Adhikari, Sunita Singh, Kshitij Karki

**Affiliations:** 1 Department of Public Health, Asian College for Advance Studies, Purbanchal University, Satdobato, Lalitpur, Nepal; 2 Research and Innovation Section, Southeast Asia Development Actions Network (SADAN), Lalitpur, Nepal; 3 Research Section, Swadesh Development Foundation (SDF), Province-2, Siraha, Nepal; 4 Department of Nutrition and Dietetics, Armed Police Force Hospital, Kathmandu, Nepal; 5 Central Department of Home Science, Padma Kanya Multiple Campus, Tribhuvan University, Kathmandu, Nepal; 6 Group for Technical Assistance, Lalitpur, Nepal; Monash University, AUSTRALIA

## Abstract

**Background:**

Postpartum depression is the most common mental health problem among women of childbearing age in resource-poor countries. Poor maternal mental health is linked with both acute and chronic negative effects on the growth and development of the child. This study aimed to assess the prevalence and factors associated with depressive symptoms among postpartum mothers in the lowland region in southern Nepal.

**Methods:**

A hospital-based analytical cross-sectional study was conducted from 1^st^ July to 25^th^ August 2019 among 415 randomly selected postpartum mothers attending the child immunization clinic at Narayani hospital. The postpartum depressive symptoms were measured using the validated Nepalese version of the Edinburg Postnatal Depression Scale (EPDS). The data were entered into EpiData software 3.1v and transferred into Stata version 14.1 (StataCorp LP, College Station, Texas) for statistical analyses. To identify the correlates, backward stepwise binary logistic regression models were performed separately for the dichotomized outcomes: the presence of postpartum depressive symptoms. The statistical significance was considered at p-value <0.05 with 95% confidence intervals (CIs).

**Results:**

Among the total 415 study participants, 33.7% (95% CI: 29.2–38.5%) of postpartum mothers had depressive symptoms. Study participant’s whose family monthly income <150 USD compared to ≥150 USD (aOR = 13.76, 95% CI: 6.54–28.95), the husband had migrated for employment compared to not migrated (aOR = 8.19, 95% CI:4.11–15.87), nearest health facility located at more than 60 minutes of walking distance (aOR = 4.52, 95% CI: 2.26–9.03), delivered their last child by cesarean section compared to normal (vaginal) delivery (aOR = 2.02, 95% CI: 1.12–3.59) and received less than four recommended antenatal care (ANC) visits (aOR = 2.28, 95% CI:1.25–4.15) had higher odds of depressive symptoms. Participants who had planned pregnancy (aOR = 0.44, 95% CI: 0.25–0.77) were associated with 56% lower odds of depressive symptoms.

**Conclusions:**

One-third of the mothers suffered from postpartum depressive symptoms. The participant’s husband migrated for employment, family income, distance to reach a health facility, delivery by cesarean section, not receiving recommended ANC visits, and plan of pregnancy were independent predictors for postpartum depressive symptoms. The study results warranted the urgency for clinical diagnosis of PPD and implementation of preventive package in study settings. Mental health education to pregnant women during ANC visits and proper counseling during the antepartum and postpartum period can also play a positive role in preventing postpartum depression.

## Background

Mental health problems among women of childbearing age (15–44 years) are neglected public health issues in resource-poor countries [1, 2]. World Health Organization (WHO) estimates depression will be the second most leading cause of disability by 2030 and contribute largely to the disease burden [3]. Though depression is common in both the male and female population, postpartum depression (PPD) is considered one of the most common mental health problem among women globally [1]. It is a non-psychotic depressive state that starts after childbirth [4, 5]. According to the *Diagnostic and Statistical Manual of Mental Disorders*, PPD is defined as a specifier for the major depressive disorder [6]. The global prevalence of postpartum depression among childbearing age women ranged from 5.0% to 74.0% [7, 8]. The burden of PPD in low-and middle-income countries is comparatively larger than in developed countries [9], but they share less than 20% of the global mental health resources [10]. Postpartum depression among women living in the resource-poor setting is often under-identified, undiagnosed, and untreated that hugely affects the health and social life of both mother and child [5]. Poor maternal mental health is linked with both acute and chronic negative effects on the growth and development of the child [11]. Children of mothers with postpartum depression are at higher risks of malnutrition, poor physical and neurocognitive outcomes, behavioral and interpersonal problems [12, 13]. The various risk factors of postpartum depression include psychological factors, obstetric risk factors, biological factors, social factors, family support, intimate partner violence, health services satisfaction during childbirth, high delivery cost, and lifestyle factors [14–18]. However, also these risk factors of PPD widely vary with the women’s age, race, ethnicity, and cultural context [19]. In Nepal, various studies reported the prevalence of PPD between 10%-30% during the period of 2002–2018 [20–22]. Nevertheless, very limited studies have explored the potential risk factors of PPD across different cultural and socio-economic contexts within Nepal.

The mother’s intense emotions, feelings, and stress are associated with the health outcomes of the infant [23]. In this context, availability, accessibility, and affordability of quality newborn health care services are key to maternal positive mental health, newborn survival, growth, and development of infants [24]. However, the large number of women in resource-poor countries like Nepal are compelled to choose high-risk birth settings due to lack of adequate skilled human resources, poor technologies, and unavailability of adequate medical and health services [25–27]. Existing literature also suggests mothers who are dissatisfied with the child care services during the perinatal period are more likely to develop a fear of childbirth and PPD symptoms which in turn affects the health and nutrition status of infants and mother’s self-care [28]. According to WHO quality of care framework for pregnant and newborn highlights various aspects of quality of care such as safe, timely, effective, efficient, equitable, and people-centered care [29, 30]. Though several studies measured the client’s satisfaction with maternal health care services, there is a lack of investigations in Nepal on postpartum depression taking newborn care services satisfaction into account. Also, a limited number of studies have assessed depressive symptoms among women living in low land (Terai region) where about half of the Nepalese population resides [31]. Therefore, this study aimed to assess the factors for the prevalence of depressive symptoms among postpartum mothers in the low land region in southern Nepal.

## Materials and methods

### Study design and setting

A hospital-based analytical cross-sectional study was conducted from 1^st^ July to 25^th^ August 2019 at Narayani hospital. The hospital is one of the oldest and high patients flow with referral tertiary care public hospitals of Nepal located in the Parsa district of Province-2 in the southern plains (Terai region) of Nepal. It lies at a distance of 135 km south of Kathmandu, the capital city of Nepal and boarder by the Bihar state of India. The hospital is one of the tertiary care hospitals in Province-2 with a 5,404,145 catchment population [31]. All the postpartum mothers who delivered their child at Narayani hospital and visited the child immunization clinic at the hospital within 10 weeks after delivery were included in the study. Mothers who had birth complications, and seriously sick, have sick children, not able to respond to questions and those who had not provided consent were excluded from the study.

### Sample size determination and sampling procedure

The sample size was calculated using the single population proportion formula N = Z^2^pq/d^2^, considering 95% confidence interval (CI), assumption of 50% proportion (p) of postpartum depression [32], 5% margin of error (d), and 10% non-response rate, the sample size was estimated to be 422. The Narayani hospital was selected purposively. A systematic random sampling technique was used to select the postpartum mothers for the study. We collected the list of 964 postpartum mothers from the immunization clinic whose babies were registered in the immunization register for receiving BCG vaccine between 10 weeks period preceding the data collection started. The sampling interval (K^th^ value) was calculated by dividing the total number of postpartum mothers by the required sample size of the study. The calculated sampling interval was two, the postpartum mother who visited the immunization clinic at first was considered as the first participant and then every postpartum mother within the interval of two visiting immunization clinic was included in the study.

### Data collection

Face-to-face interviews were conducted using a structured and validated questionnaire in the local language to collect the data. The data collectors were nursing graduates and field supervisors were public health graduates who were fluent in the local language. Four days of training were provided to surveyors on data collection, sample selection, study tools administration, and data handling procedures. All the surveyors were familiar with the objectives, methods, and ethical aspects of the study. The quality of the data was ensured by field supervisors through cross-verification of the filled questionnaire on-site, and any discrepancies and/or missing data were re-collected. Study tools were translated into Nepali language and pretested among ten percent of the study sample (n = 42) in the postnatal clinic of a similar hospital in the province-2 before the tools were used for data collection. The necessary modifications of socio-demographic and gynecological characteristics-related questions were made for clarity, understanding, suitability, and formatting after the pretesting of the tools.

### Outcome variable

#### Postpartum depressive symptoms

Postpartum depressive symptom was measured using the Edinburg Postnatal Depression Scale (EPDS) [33]. Previous studies have used a cut-off point of ≥10 with high sensitivity and specificity to rule out the depressive symptoms among postpartum mothers [21, 34]. Therefore, the cut of EPDS score ≥10 was used to assess the postnatal depressive symptoms among the participants of this study, which has a sensitivity of 91% and specificity of 84% [35]. The EPDS has 10 items of questionnaires for the assessment of depressive symptoms. The responses in each question scored 0–3. The total possible score after addition ranged from 0–30, with a higher score indicating a higher level of depressive symptoms. Also, the previous studies suggested that cut off levels of EPDS score ≥10 can reduce the detection errors of depressive symptoms among postpartum mothers [33, 34]. In Nepal, the validation of the Nepalese version of the EPDS study showed good validity and recommended to use in Nepal for the screening of PPD [36]. Cronbach’s alpha for the EPDS scores in this study was 0.76.

### Predictor variables

The predictor variables included the socio-demographic, obstetric, and newborn care services satisfaction variables.

#### Socio-demographic and obstetric variables

The socio-demographic information of the study participants such as age, age at marriage, ethnicity, education status, type of family, employment status, employment status of respondent’s partner, husband migration status, family monthly income, distance to reach the nearest health facility, family support during pregnancy and delivery, and sex of child were collected through a structured questionnaire. The study respondent’s ethnicity was categorized into advantaged ethnic groups and disadvantaged ethnic groups for further analysis [37]. The family type was categorized into nuclear (couple living with their children under the age of eighteen years) and joint or extended family (couples living with their children under the age of eighteen years and older parents in the same household). The migration was considered if the participant’s male partner had migrated within or outside the country for employment purposes in the last 6 months before their last childbirth and absence during the time of delivery and postnatal period. Similarly, respondent’s obstetric related information such as parity, plan of pregnancy, type of delivery, times of antenatal care (ANC) visits, times of postnatal care (PNC) visits were also collected through structured questionnaires. The type of delivery was categorized into normal or vaginal assisted and cesarean section.

#### Newborn care services satisfaction

Newborn care services satisfaction among postnatal mothers was assessed using the 11 items satisfaction scale which was also used by Nepal Health Facility Survey (NHFS) [38, 39]. This health services satisfaction scale is constructed based on the WHO Quality of care framework [29, 30]. The 11 items service satisfaction scale included eleven statements concerning to problem encountered by postpartum mother during the health facility visit for the newborn care services: 1) time waited to see providers, 2) ability to discuss problems, 3) amount of explanation received on the problem or treatment, 4) privacy from having others hear the consultation, 5) privacy from having other to see the consultation, 6) availability of medicine, 7) opening and closing time of health facility, 8) the number of days services availability, 9) cleanliness status of health facility, 10) satisfaction with the behavior of staff, and 11) cost for the newborn care services. Postpartum mothers who encountered problems on these statements were categorized as “yes” (coded = 1) and not encountered problems for the statement was categorized as “no” (coded = 0). The total possible score ranged from 0–11. The score equals or above the median value was defined as “not satisfied” and below the median value defined as “satisfied” with the newborn care services received at the health facility. Thus, in our analysis, the satisfaction status was categorized to form one of the predictor variables (satisfied versus unsatisfied) considering the median score as a cutoff point.

### Data processing and statistical analysis

The collected data were entered into EpiData software 3.1v and transferred into Stata version 14.1 (StataCorp LP, College Station, Texas) for statistical analyses. The descriptive results are presented in the form of mean, standard deviation, frequency, and percentage. To identify the correlates, binary logistic regression models were performed separately for the dichotomized outcomes: the presence of postpartum depressive symptoms. Independent variables significant at or < 0.05 in the unadjusted models were adjusted in the backward stepwise logistic regression model. Multicollinearity amongst the predictor variables was checked using the Variance Inflation Factor (VIF) considering the cutoff point value <3 [40]. The statistical significance was considered at p-value <0.05 and 95% confidence intervals (CIs).

### Ethical considerations

Ethical approval for this study was received from the Ethical Review Board (ERB) of Nepal Health Research Council (Reg. No: 679/2018). The Narayani hospital also provided formal approval to conduct this study. Written consent was obtained from each of the study participants before the interview was conducted. The study participants were informed about voluntary participation, their right to withdraw from the interview at any point, and the confidentiality of the data provided.

## Results

### Socio-demographic characteristics of the respondents

Of the total 422 interviewed postpartum mothers, 415 mothers provided complete information which made the response rate of 98.3%. Among the respondents, 311 (74.9%) were below 25 years of age with a mean (± SD) age of the respondents 22.2 ± 3.8 years. Mostly, participants were from advantage ethnic group (58.1%) and the majority of the respondents lived in a joint/extended family 292 (70.4%). The majority of the respondents were literate 235 (56.6%) and more than half of the respondents were unemployed 217 (52.3%). More than one-third of the respondent’s husbands 155 (37.3%) were migrated for employment. The average (± SD) monthly income of the household was USD 146.19 ± SD 55.8. The mean (± SD) age at marriage for the respondents was 18.4 years (±1.7) and more than half of the women 213 (51.3%) were first-time moms. About 148 (35.7%) of the respondents’ delivery was conducted with cesarean section. Similarly, about 125 (30.1%) women had less than four ANC visits and about 266 (64.1%) of mothers had not attended recommended PNC visits. Slightly less than half (46%) of the postpartum mothers were unsatisfied with the newborn care services (Table 1).

**Table 1:** Socio-demographic information of the postpartum women (n = 415).

Variables	Frequency (n)	Percentage (%)
**Mean age (years) (Mean ± SD)**	22.23	3.8
**Age at marriage (years) (Mean ± SD)**	18.40	1.7
**Family monthly income (USD) (Mean ± SD)**	146.19	55.8
**Respondent age (years)**		
<25 years	311	74.9
≥25 years	104	25.1
**Parity**		
Primiparous	213	51.3
Multiparous	202	48.7
**Sex of last-child**		
Male	211	50.8
Female	204	49.2
**Education status**		
Illiterate	180	43.4
Literate	235	56.6
**Ethnicity**		
Advantage ethnic group	241	58.1
Disadvantage ethnic group	174	41.9
**Type of family**		
Nuclear	123	29.6
Joint/extended	292	70.4
**Employment status of respondents**		
Yes	198	47.7
No	217	52.3
**Employment status of respondents partner**		
Yes	280	67.5
No	135	32.5
**Husband migration for employment**		
Yes	155	37.3
No	260	62.7
**Plan of pregnancy**		
Yes	237	57.1
No	178	42.9
**Type of delivery**		
Normal or Vaginal Assisted	267	64.3
Cesarean section	148	35.7
**Family support during pregnancy and delivery**		
Yes	326	78.6
No	89	21.4
**History of intimate partner violence during pregnancy**		
Yes	98	23.6
No	317	76.4
**ANC visit during last pregnancy**		
< 4 ANC visit	125	30.1
≥ 4 ANC visit	290	69.9
**Completed recommended PNC visit**		
Yes	149	35.9
No	266	64.1
**Distance to reach the nearest health facility**		
<30 minutes	196	47.2
30–60 minutes	118	28.4
>60 minutes	101	24.3
**Family income (USD)**		
<150 USD	255	61.4
>150 USD	160	38.5
**Respectful treatment**		
No	228	54.9
Yes	187	45.1
**Explanation about treatment**		
No	170	40.9
Yes	245	59
**Language barrier**		
No	196	47.2
Yes	219	52.7
**Newborn care satisfaction score (median, interquartile range)**	5	(4, 8)
**Newborn care satisfaction**^a^		
Satisfied	223	53.7
Unsatisfied	192	46.2
**Depressive symptoms score (Mean ± SD)**	6.39	4.3
**Depressive symptoms**		
No depressive (EPDS score <10)	275	66.2
Depressive (EPDS score ≥10)	140	33.7

^a^median split to formed the dichotomized variable from newborn care satisfaction score

SD: standard deviation

USD $1 = NRs.107.0 (Nepalese currency)

### Prevalence of postpartum depressive symptoms

**Fig 1:**
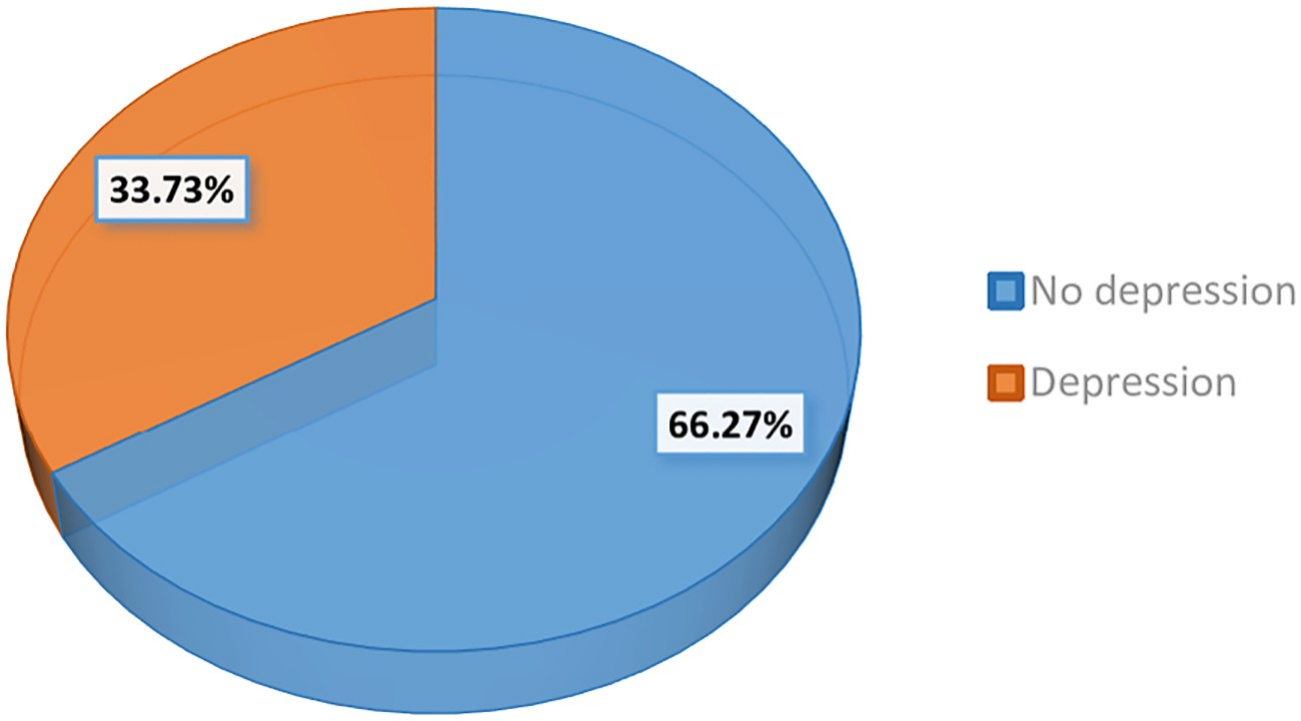
Prevalence of depressive symptoms among postpartum mothers.

**Fig 2:**
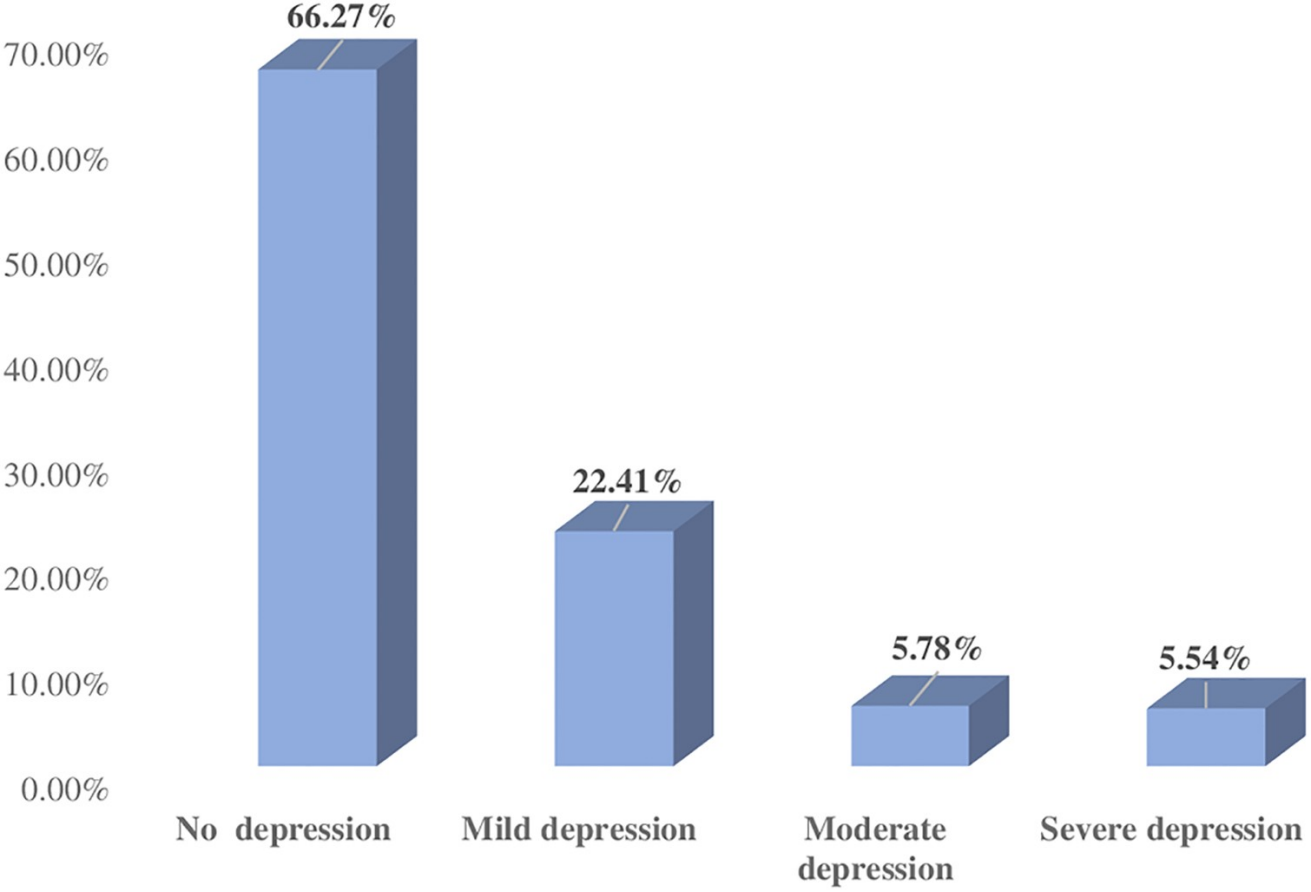
Classification of postpartum depressive symptoms according to the severity.

Out of 415 postpartum mothers, 33.7% (95% CI: 29.2–38.5%) of mothers had depressive symptoms (Fig 1). The prevalence of mild, moderate, and severe depressive symptoms among mothers were 22.4%, 5.7%, and 5.5% respectively (Fig 2).

### Determining factors of postpartum depressive symptoms

In bivariate analyses, mothers’ ethnicity, education status, sex of last-child, family structure, husband migration for employment, plan of pregnancy, type of delivery, ANC visit during last pregnancy, PNC visit during last delivery, family support during pregnancy, distance to reach a nearest health facility, family monthly income, and unsatisfied with the newborn care services were significantly associated with higher odds of depressive symptoms of postpartum mothers (Table 2). In the stepwise backward multivariate logistic regression, several factors were significantly higher odds of depressive symptoms (Table 2): mothers who had family monthly income <150 USD compared to ≥150 USD (aOR = 13.76, 95% CI: 6.54–28.95), mothers whose husband were migrated for employment compared to not migrated for employment (aOR = 8.19, 95% CI:4.11–15.87), mothers whose nearest health facility was at more than 60 minutes walking distance compared to less than 30 minutes (aOR = 4.52, 95% CI: 2.26–9.03), Postpartum mothers who had delivered their last child by cesarean section compared to normal (vaginal) delivery (aOR = 2.02, 95% CI: 1.12–3.65), and receiving less than the recommended ANC visits (aOR = 2.28, 95% CI:1.25–4.15). On the other hand, mothers who had planned pregnancy (aOR = 0.44, 95% CI: 0.25–0.77) were associated with 56% lower odds of depressive symptoms.

**Table 2:** Bivariate and multivariate logistic regression for factors associated with depressive symptoms among postpartum mothers (n = 415).

Variables	Postpartum depressive symptoms		Bivariate analysis	Multivariate analysis
	No (EPDS score <10)	Yes (EPDS score ≥10)	cOR (95% CI)	aOR (95% CI)
	n (%)	n (%)		
**Respondent age (years)**				
<25 years	205 (65.9)	106 (34.1)	Ref	
≥25 years	70 (67.3)	34 (32.9)	0.94 (0.58–1.50)	
**Parity**				
Primiparous	145 (68.1)	68 (31.9)	Ref	
Multiparous	130 (64.6)	72 (35.4)	1.18 (0.78–1.77)	
**Sex of child**				
Male	155 (73.6)	56 (26.4)	Ref	Ref
Female	120 (58.8)	84 (41.2)	1.94 (1.28–2.93)**	1.52 (0.86–2.70)
**Education status**				
Illiterate	96 (53.3)	84 (46.7)	2.79 (1.83–4.25)***	-
Literate	179 (76.7)	56 (23.3)	Ref	
**Ethnicity**				
Advantage ethnic group	168 (69.1)	73 (30.9)	Ref	1.16 (0.66–2.04)
Disadvantage ethnic group	107 (61.9)	67 (38.1)	1.44 (0.95–2.17)	
**Type of family**				
Nuclear	71 (57.7)	52(42.3)	Ref	Ref
Joint/extended	204 (69.8)	88 (30.2)	1.69 (1.09–2.63)**	0.83 (0.44–1.55)
**Employment status of respondents**				
Yes	96(48.8)	102(51.2)	Ref	-
No	179(82.9)	38(17.1)	5.00 (3.19–7.83)***	
**Employment status of respondents partner**				
Yes	176 (62.6)	104 (37.4)	Ref	-
No	99 (73.3)	36 (26.7)	1.63 (1.03–2.55)*	
**Husband migration for employment**				
Yes	73 (47.1)	82 (52.9)	3.91 (2.54–6.01)***	8.19 (4.11–15.87)***
No	202 (77.9)	58 (22.1)	Ref	Ref
**Plan of pregnancy**				
Yes	174 (73.4)	63 (26.6)	0.47 (0.31–0.72)***	0.44 (0.25–0.77)**
No	101 (56.4)	77(43.6)	Ref	Ref
**Type of delivery**				
Cesarean section	79 (53.8)	69 (46.2)	Ref	2.02 (1.12–3.59)
Normal	196 (73.1)	71 (26.9)	2.41 (1.58–3.68)***	Ref
**Family support during pregnancy and delivery**				
Yes	226 (69.3)	100 (30.6)	Ref	-
No	49 (55.1)	40 (44.9)	1.84 (1.14–2.98)**	
**intimate partner violence during pregnancy**				
Yes	50(51.2)	48 (48.8)	2.35 (1.48–3.73)***	-
No	225 (70.8)	92 (29.2)	Ref	
**ANC visit during last pregnancy**				
< 4 ANC visit	66 (52.8)	59 (47.2)	2.31 (1.49–3.56)***	2.28 (1.25–4.15)**
≥ 4 ANC visit	209 (72.1)	81 (27.9)	Ref	Ref
**Completed recommended PNC visit**				
Yes	114 (76.1)	35 (23.9)	Ref	Ref
No	161 (60.3)	105 (39.7)	2.12 (1.35–3.34)**	1.61 (0.88–2.94)
**Distance to reach nearest health facility**				
<30 minutes	158 (80.1)	38 (19.9)	Ref	Ref
30–60 minutes	76 (64.1)	42 (35.9)	2.29 (1.37–3.85)**	2.23 (1.15–4.35)*
>60 minutes	41 (40.9)	60 (59.9)	6.08 (3.57–10.35)***	4.52 (2.26–9.03)***
Family income (USD)				
<150 USD	132 (51.7)	123 (48.3)	7.83 (4.48–13.72)***	13.76 (6.54–28.95)***
≥150 USD	143 (89.3)	17 (10.7)	Ref	Ref
**Newborn care satisfaction**				
Satisfied	172 (77.1)	51 (22.93)	Ref	Ref
Unsatisfied	103 (53.6)	89 (46.4)	2.91 (1.91–4.44)***	1.69 (0.95–3.01)

*p<0.05,

**p<0.02,

***p<0.001, cOR; crude odds ratio for unadjusted logistic regression model, aOR; adjusted odds ratio for backward stepwise logistic regression model,

In multivariate analysis, the employment status of the respondent, respondent partner employment, respondent education, family-supportive during pregnancy, and intimate partner violence are omitted due to the collinearity problem.

## Discussion

In this study, postpartum depressive symptoms were found among one-third of mothers which accounted for 33.7% (95% CI: 29.2–38.5%). This finding is consistent with previous studies undertaken in a different time in different areas, where the prevalence of PPD among mothers during this course were 30.3% in Paropakar Maternity and Women’s Hospital (PMWH), Kathmandu, Nepal [21], 34.6% in South-East Nigeria [21], about 39.4% in urban slums of Dhaka, Bangladesh [16] and 34.6% in Karachi, Pakistan [41] using EPDS score. In Nepal, Several studies reported the prevalence of postpartum depressive symptoms among mothers were ranging from 10–30% during different periods in the different settings using an EPDS score cut-off ≥10–13 [20–22]. The prevalence of PPD from the current study is slightly higher than the other three studies conducted at different hospitals in Nepal, where 22.7% in Tribhuvan University Teaching Hospital (TUTH), Kathmandu, Nepal [22], 19.4% in Kathmandu Medical College Teaching Hospital (KMCTH), and 22.2% in Dhading District Hospital, Nepal [42]. However, all three studies had used an EPDS score >12 as a cut-off point. In this study, a relatively high prevalence of PPD was observed due to the lower cut-off points of EPDS score ≥10 than that of other studies from Nepal. These findings are in line with the previous study from Nepal, where the prevalence of PPD was 30.3% using a cut-off point ≥10 [21]. Other reasons might be due to the difference in study settings, study year, socio-demographic and topographical structures. The estimates of our findings are in line with the results from the systematic review and meta-analysis done in low-and middle-income countries that showed the prevalence of PPD between 11 to 40% [43]. The current study revealed that several factors were significantly associated with higher odds of PPD among mothers in the adjusted multivariate model: husband migration for employment, type of delivery, mothers ANC visits during pregnancy, distance to reach the nearest health facility center family income.

This study found that mothers who belong to low-income families (<150 USD/month family income) were significantly associated with depressive symptoms. These findings are supported by the evidence from a systematic review undertaken in 17 low-and middle-income countries, where mothers who had low income or financial difficulties were more likely to have PPD [43]. Financial problems and low-income sources among women before or during pregnancy may affect the mental health of mothers during postpartum periods [44]. Likewise, various studies have reported that the low socioeconomic factor constitutes strong risk factors for depressive symptoms in postpartum mothers [45–47].

Our study revealed that PPD was higher among mothers who have unplanned pregnancies. This result was congruent with similar studies conducted in Bangladesh and Malaysia [16, 48]. It could be due to the reason that unplanned pregnancies are most often associated with poor maternal wellbeing that may develop long-term adverse psychological consequences [49]. Also, inadequate birth preparedness and poor stress coping mechanisms particularly among socio-economic disadvantaged women may exacerbate the mental health problems during the postpartum period [50, 51]. Therefore, screening during ANC visits to identify high-risk groups would be crucial to prevent PPD.

The current study reported the burden of PPD was higher among those participants whose husband was migrated for employment within the last 6 months before their last child was born. The possible reason could be that they have not had their intimate person to share the emotions, feelings, and face the difficult circumstances which may arise during the postpartum period. They may also feel alone or lack of support that they expected to receive from their spouse. Thus, suppression of the mother’s feelings and emotions can aggregate the depressive symptoms during the postpartum period [22]. Love, caring and emotional support by their husband may enhance the self-esteem and helps build confidence during the most critical situation [22].

In the present study, PPD symptoms were seen to be associated with mothers whose deliveries were conducted by cesarean section. Risk factors for postnatal depressive symptoms were similar to those for antenatal depressive symptoms, as well as assisted delivery [52]. Considering this evidence, our results are consistent with Bitew et al. findings where antenatal depression was associated with assisted delivery and uptake of unplanned institutional delivery which increased the risk of emergencies related to labor complications resulting cesarean section delivery [53]. Likewise, another study also supported this evidence, where depression was associated with prolonged labor, and other perinatal complications [54, 55].

This study found that the PPD symptoms were positively associated with mothers who visited <4 times ANC during their pregnancy period. This might happened because mothers who did not complete the recommended four ANC visits would not get proper counseling on different aspects of the problem during the pregnancy periods. Mothers who were in regular ANC visits could get consistent counseling and support from the health professionals which helped them to enhance the self-confidence and self-esteem [16]. This indicates the high importance of educating pregnant women about postpartum psychological changes during the time of ANC visits that may help them for self-identification of early signs and symptoms of PPD [56].

In the current study, PPD was likely to be higher among mothers who live at more than one-hour walking distance from the nearest health facility. This result is supported by the findings from the studies conducted in South Africa and Canada where depressive symptoms among participants were positively associated with increasing distance to reach the nearest health facility [57, 58]. This might be due to the reason that people whose residence is at a far distance from healthy facility lives in greater isolation and have difficulty in reaching health facility for receiving the health services. In this study, the positive association of PPD with mothers who did not have formal education was found in bivariate analysis, but the association could not assess in the adjusted model due to the multicollinearity problem. These findings are consistent with Agbaje et al. who reported the low level of education is a strong risk factor for PPD in women [59]. Depressive symptoms were seen in unemployed women in the bivariate analysis, however, the association could not analyze in the adjusted model due to the multicollinearity problem. In contrast, these findings are inconsistent with Azad R et al. results from Bangladesh who reported that the PPD was higher among those mothers who were employed but quit the job due to pregnancy [16]. In the current study, Intimate partner violence was positively associated with PPD among mothers in bivariate analysis, but not found significant in multivariate regression analysis. Several studies reported the intimate partner violence (IPV) is one of the most important risk factors for developing postpartum depression among mothers [16, 60, 61]. According to the Nepal Demographic and Health Survey (NDHS) 2016, about 26% of married women had experienced intimate partner violence in Nepal and a high number (34%) in Province 2 [62], where the current study was conducted. This might be due to the reason that more than one-third of the participants’ husbands in our study were migrated for employment. Also, those who were living together may felt hesitant to express experiences of intimate partner violence openly in Nepalese male dominants society [63].

The present study had some limitations. Firstly, this study design was cross-sectional, thus we could not identify the casual pathway for postpartum depressive symptoms among mothers. Secondly, the sample included in this study was mothers who were visiting the tertiary care hospital for immunization for her baby. Therefore, our sample did not include mothers who were going to other health facilities such as health posts and primary health care centers where immunization services are provided. So, these findings limiting the generalizability to all levels of health facility users. Thirdly, the study used an EPDS questionnaire as a screening tool for measuring depression symptoms which was not a clinical diagnosis. Also, genetic factors and lifestyle factors were not included to measure predictors for PPD. Despite these limitations, our study had some strengths. To the best of our knowledge, this study is the first to explore the postpartum depressive symptoms among mothers and associated factors in the context of the lowland region in the southern plain (Terai region) of Nepal where the majority of the women have resided with low socioeconomic status. We used the repeatedly validated pretested Nepali version tools and scale which was nationally and internationally recognized and validated for use. Our study suggested the community-based screening of PPD and its clinical confirmation. Further, the study results suggest the urgent need for designing, implementing, and evaluating preventive packages.

## Conclusions

The study results showed one in every three mothers had postpartum depressive symptoms. Likewise, the husband migrated for employment, family income, distance to reach a health facility, delivery by cesarean section, not receiving recommended ANC visits were independent predictors for postpartum depressive symptoms. Mental health education to pregnant women during ANC visits can play a positive role in preventing postpartum depression. Adequate counseling during delivery and family support during delivery are also crucial factors in preventing postpartum depression among mothers. Likewise, the results suggest maternal and child health programs should also integrate maternal mental health component.

## Supporting information

S1 Raw data (SAV)

## Abbreviations

ANC: Antenatal Care
aOR: Adjusted Odds Ratio
cOR: Crude odds Ratio
EPDS: Edinburg Postnatal Depression Scale
PNC: Postnatal Care
PPD: Postpartum Depressive
